# RpoS Plays a Central Role in the SOS Induction by Sub-Lethal Aminoglycoside Concentrations in *Vibrio cholerae*

**DOI:** 10.1371/journal.pgen.1003421

**Published:** 2013-04-11

**Authors:** Zeynep Baharoglu, Evelyne Krin, Didier Mazel

**Affiliations:** 1 Institut Pasteur, Unité Plasticité du Génome Bactérien, Département Génomes et Génétique, Paris, France; 2 CNRS, UMR3525, Paris, France; Uppsala University, Sweden

## Abstract

Bacteria encounter sub-inhibitory concentrations of antibiotics in various niches, where these low doses play a key role for antibiotic resistance selection. However, the physiological effects of these sub-lethal concentrations and their observed connection to the cellular mechanisms generating genetic diversification are still poorly understood. It is known that, unlike for the model bacterium *Escherichia coli*, sub-minimal inhibitory concentrations (sub-MIC) of aminoglycosides (AGs) induce the SOS response in *Vibrio cholerae*. SOS is induced upon DNA damage, and since AGs do not directly target DNA, we addressed two issues in this study: how sub-MIC AGs induce SOS in *V. cholerae* and why they do not do so in *E. coli*. We found that when bacteria are grown with tobramycin at a concentration 100-fold below the MIC, intracellular reactive oxygen species strongly increase in *V. cholerae* but not in *E. coli*. Using flow cytometry and *gfp* fusions with the SOS regulated promoter of *intIA*, we followed AG-dependent SOS induction. Testing the different mutation repair pathways, we found that over-expression of the base excision repair (BER) pathway protein MutY relieved this SOS induction in *V. cholerae*, suggesting a role for oxidized guanine in AG-mediated indirect DNA damage. As a corollary, we established that a BER pathway deficient *E. coli* strain induces SOS in response to sub-MIC AGs. We finally demonstrate that the RpoS general stress regulator prevents oxidative stress-mediated DNA damage formation in *E. coli*. We further show that AG-mediated SOS induction is conserved among the distantly related Gram negative pathogens *Klebsiella pneumoniae* and *Photorhabdus luminescens*, suggesting that *E. coli* is more of an exception than a paradigm for the physiological response to antibiotics sub-MIC.

## Author Summary

Bacteria can remodel their genome in order to adapt to new environments. This genomic plasticity can be mediated by horizontal gene transfer, genome rearrangements, and mutations. One important factor is the bacterial SOS response, induced upon DNA damaging stress conditions. SOS response induction leads to increased mutation frequencies and genome rearrangements. Antibiotics such as fluoroquinolones cause direct DNA damage and induce the SOS response. Other antibiotics such as aminoglycosides (AGs) do not target DNA and do not induce SOS in *E. coli*. However, it was recently shown that low concentrations of AGs, which do not inhibit bacterial growth, do induce SOS in *V. cholerae*. We show here that AG-dependent SOS induction is caused by oxidative stress in *V. cholerae* and that *E. coli* is well protected against this kind of oxidative insult thanks to efficient oxidative stress response through the RpoS general stress response sigma factor. We propose that the model bacterium *E. coli* is not a paradigm in this case, as other pathogens such as *Klebsiella pneumoniae* and *Photorhabdus luminescens* also induce SOS in response to sub-MIC AGs.

## Introduction

*Vibrio cholerae* is a severe human pathogen growing on crustacean shells or planktonically in the aquatic environment. *V. cholerae* couples environmental stress and adaptation through SOS response dependent gene expression and mutagenesis, induced, for instance, during ssDNA uptake [1]–[2] or after antibiotic treatment [3]. The SOS stress response occurs through the induction of an entire regulon controlled by the LexA repressor [4]. In the presence of abnormal levels of single stranded DNA (ssDNA) in the cell, RecA, the pivotal protein of homologous recombination, forms a nucleofilament on ssDNA which catalyses the self-cleavage of the LexA repressor. Inactivation of LexA releases the transcription of recombination and repair genes belonging to the SOS regulon. ssDNA levels increase in the cell during horizontal gene transfer but also when replication is blocked in the presence of any DNA damaging agent such as antibiotics that target DNA (like fluoroquinolones or mitomycin C) or UV irradiation.

We recently reported the effects on the SOS response induction of sub-Minimal Inhibitory Concentrations (sub-MIC) of antibiotics from different families in *V. cholerae* [3]. Sub-MIC of antibiotics may be found in several environments [5], in particular in the mammalian hosts of pathogenic and commensal bacteria, where they can play a very important role for the selection of resistant bacteria [6]. Moreover, a large part of the ingested antibiotics is rejected unchanged in the environment [7]–8. Unlike above-MIC, the biological effects of sub-MIC of antibiotics have not been studied in detail. Transcriptome and proteome analyses have shown that many antibiotics exhibit contrasting properties when tested at low compared to high concentrations: it has been noted that sub-MIC of antibiotics induce several changes in expression profiles of a wide range of genes unrelated to the target function [9]. Sub-MIC of antibiotics induce phenotypic changes, including an increase in mutagenesis [3]. Strikingly, we observed that sub-MIC of aminoglycosides (referred to as AGs throughout this manuscript), chloramphenicol, rifampicin and tetracycline induce SOS in *V. cholerae* [3]. These antibiotics do not directly target DNA synthesis or the DNA molecule and they do not induce SOS in *E. coli* [3]. The fact that they induce SOS in *V. cholerae* suggests a role for intermediate factors that cause stress and lead to DNA damage in this bacterium. A recent study demonstrated how beta-lactams, fluoroquinolones (FQs) and AGs stimulate production of reactive oxygen species (ROS) in bacteria [10]. ROS can damage DNA and proteins, and induce mutagenesis, increasing as such the odds of resistance conferring mutations, ultimately leading to multiple resistances.

DNA damage can indeed be caused by ROS [11]: DNA is the target of hydroxyl radicals (OH^−^) and the Fenton reaction is the major source of OH^−^ formation [12] in the presence of iron ions [13]–[14]. Iron can localize along the phosphodiester backbone of nucleic acids and OH^−^ attacks DNA sugar and bases and ultimately causes double strand breaks, which are repaired by the RecBCD homologous recombination pathway [15].

Another type of DNA damage caused by oxidative attack is the incorporation of oxidized guanine residues (7,8-Dihydro-8-oxo-guanine or 8-oxo-G). *E. coli* and *V. cholerae* possess a defense system against 8-oxo-G which involves MutT, MutY and MutM [16]. MutT hydrolyses 8-oxo-G in the nucleotide pool, whereas MutY and MutM limit incorporation of 8-oxo-G and mismatch formation [17]. Here too, incomplete action of this base excision repair system may lead to double strand DNA breaks that are cytotoxic if unrepaired [18].

On the other hand, it is known that mistranslated and misfolded proteins are more susceptible to oxidation [19]. Moreover, mistranslational corruption of proteins may lead to replication fork collapse and induction of SOS [20]. It has been shown in *Deinococcus radiodurans* that cell death by radiation is not caused by direct DNA damage but primarily by oxidative damage on proteins, which eventually results in the loss of DNA repair [21]. Proteome protection against ROS thus seems to be at least as important as DNA protection for keeping cell integrity in times of oxidation. Knowing that AGs target protein translation, it was appealing to determine if sub-MIC of these antibiotics induce ROS formation in *V. cholerae* and to try to characterize mechanisms involved in the induction of SOS.

Finally, oxidative stress is known to induce the RpoS regulon [22]–[23]. RpoS is the stationary phase sigma factor, which is induced in exponential phase in response to stress [24]–[26]. Genes expressed following RpoS regulon induction, namely catalases (KatE, KatG) and iron chelators (Dps), protect cells from ROS related DNA damage [27], e.g. double strand DNA breaks caused by hydroxyl radicals generated through the Fenton reaction [12]–[13], [28]. It has been shown in *Vibrio vulnificus* (a human pathogen) that RpoS is essential in exponential growth phase to overcome H_2_O_2_ related oxidative stress [29]. Moreover, it was observed that *V. vulnificus* is more sensitive to H_2_O_2_ than *E. coli* and that KatG catalase expression is more strongly reduced in a *ΔrpoS* mutant in *V. vulnificus* compared to *E. coli*. These observations pointed to an effect of RpoS in the differences on the response to H_2_O_2_ between these two species, and as *V. cholerae* is more phylogenetically related to *V. vulnificus* than to *E. coli*, one would expect more conservation for the RpoS response between the two *Vibrio* species.

RpoS levels are regulated at several stages in *E. coli*: (i) transcription [30] (ii) mRNA stability [31] and (iii) protein stability. Indeed, the RpoS protein is targeted to the ClpXP protease for degradation by the adaptor protein RssB [32]–[34]. *ΔclpP* mutants accumulate RpoS [34]. Anti-adaptors IraD, IraM, IraP are induced following stress (respectively: oxidative stress, magnesium and phosphate starvation) [32] and prevent this targeting by RssB, thus stabilizing the RpoS protein [33]. A *ΔrssB* mutant relieves H_2_O_2_ sensitivity of an *E. coli ΔiraD* mutant in an RpoS dependent fashion. Interestingly, the IraD anti-adaptor, which is induced during oxidative stress and DNA damage is conserved only in *E. coli* and *Salmonella* and is absent from *V. cholerae* [24]–[25], whereas RssB is present in *V. cholerae*, suggesting that *E. coli* RpoS is more effectively protected from degradation during stress than *V. cholerae* RpoS. Moreover, among Gram negative pathogens, *E. coli* and *Salmonella* seem to be the only species that carry these anti-adaptors, suggesting that other pathogens may behave like *V. cholerae* in terms of RpoS degradation.

Here, we test and show that sub-MIC of the AG tobramycin induces oxidative stress in *V. cholerae*. Moreover, we show that this SOS induction is mostly due to hydroxyl radical formation and 8-oxo-G incorporation in DNA using GFP as a reporter of SOS thanks to fusions of *gfp* with the SOS-dependent *intIA* promoter constructed for our previous studies [1], [3]. Finally, we provide evidence for a role of RpoS in the protection of *E. coli* and *V. cholerae* cells against sub-MIC tobramycin induced stress and propose that most of the induction is linked to the rapid degradation of the RpoS protein in *V. cholerae*. We further show that the SOS induction by AGs is conserved among distantly related Gram negative pathogens.

## Results

### Oxidative stress and ROS formation is induced by tobramycin in *V. cholerae*

In order to understand how sub-MIC of antibiotics such as AGs that do not directly target DNA (AGs) impact the bacterial cell and induce a stress response in *V. cholerae*, we aimed at determining if SOS induction by sub-MIC takes place following oxidative stress.

**Figure 1:**
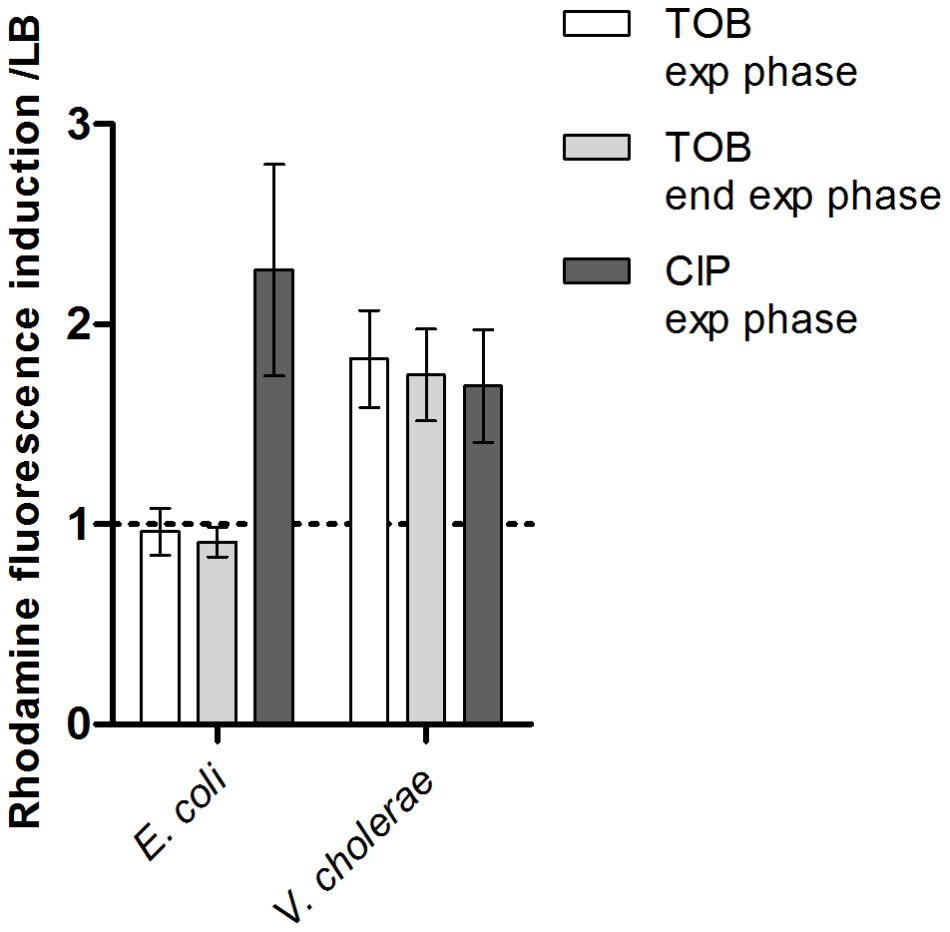
Sub-MIC tobramycin treatment induces ROS formation in *V.*cholerae and not in *E. coli*. Histogram bars show the ratio of rhodamine fluorescence in the presence of the specified antibiotic over fluorescence in LB and thus reflect the induction of ROS formation. Tobramycin (TOB) and ciprofloxacin (CIP) concentrations were respectively 0.01 µg/ml and 0.005 µg/ml for *V. cholerae*, and 0.1 µg/ml and 0.05 µg/ml for *E. coli*. Exp phase stands for exponential phase i.e. an OD600 nm of 0.5. End exp phase stands for end of exponential phase i.e. an OD600 nm of 0.8.

First we addressed whether there is a difference in ROS formation between *E. coli* and *V. cholerae*. We chose to use low doses of tobramycin (100 fold below the MIC) in both bacteria because this antibiotic was used in previous studies [3]. These concentrations (0.1 µg/ml for *E. coli* and 0.01 µg/ml for *V. cholerae*) were previously shown to induce SOS in *V. cholerae* and not in *E. coli* [3]. We performed growth in LB medium with and without tobramycin in the presence of dihydrorhodamine 123 (DHR), a chemical agent that becomes fluorescent upon oxidation into rhodamine 123 in the presence of intracellular ROS [35]. DHR oxidation actually reports the presence of H_2_O_2_ and intracellular peroxidases [36], and peroxidases are induced in response to oxidative stress and catalyze the formation of ROS. Fluorescence is thus interpreted as peroxidase induction and generation of free oxygen radicals [36]. We measured the intracellular generation of free radicals/peroxidase induction through rhodamine fluorescence at the middle and end of exponential phase (OD600 0.5 and 0.8). Our results showed that, when these bacteria were grown in the presence of tobramycin at 1/100 MIC, peroxidase induction (i.e. oxidative stress) took place in *V. cholerae* but not in *E. coli* (Figure 1). Ciprofloxacin (a fluoroquinolone) was used as a control known to induce ROS formation in *E. coli* [10]. Ciprofloxacin also induced SOS in both bacteria at sub-MIC (at a concentration of 1/100 of the MIC) [3]. As expected, sub-MIC of ciprofloxacin induced DHR oxidation in *E. coli* and in *V. cholerae*. These results point to a significant activation of the oxidative stress response and ROS formation in *V. cholerae* in response to sub-MIC tobramycin treatment.

### Hydroxyl radicals and incorporation of 8-oxo-G during growth of *V. cholerae* on tobramycin leads to SOS induction

**Figure 2:**
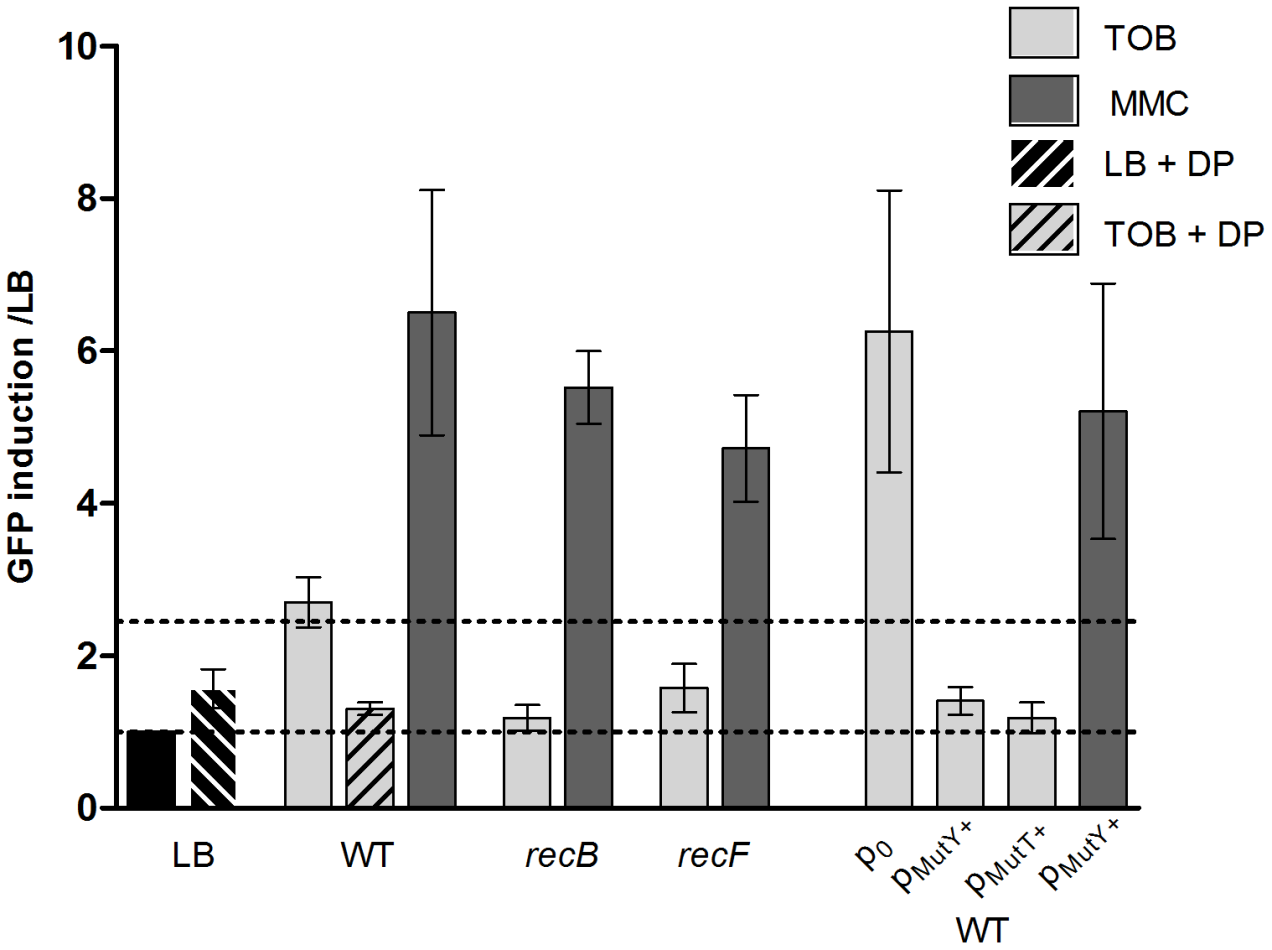
Factors modulating SOS induction by tobramycin in *V.*cholerae. Histogram bars show the ratio of GFP fluorescence in a given strain in the presence of antibiotic over fluorescence of the same strain grown in LB and thus reflect the induction of SOS by the antibiotic. p0 stands for empty pTOPO plasmid. pMutY+ stands for pTOPO-MutY+ (p9476). TOB: tobramycin 0.01 µg/ml. MMC: mitomycin C 0.1 µg/ml. DP: 2,2 Dipyridyl 0.5 mM.

In order to study the nature of DNA damage caused by sub-MIC tobramycin, we alternatively inactivated the RecFOR gap repair and RecBCD double strand break repair pathways by deleting *recF* and *recB* respectively in the wild type *V. cholerae* strain (Figure 2). We used GFP fused to the *intIA* promoter as the SOS reporter (plasmid p4640 for *V. cholerae*) [1], [3]. Mitomycin C (MMC) cross-links the two DNA strands, leading to double strand break formation, which induces SOS. MMC was thus used as an SOS inducer and tested as a positive control. The basal GFP fluorescence on LB was at the same level for all strains shown in Figure 2. The RecFOR pathway is involved in the induction of SOS by allowing RecA nucleo-filament formation on single strand DNA lesions, while the RecBCD pathway recruits RecA on double strand DNA breaks [15]. SOS induction following tobramycin treatment decreased from 3 fold for wild type to 1.2 fold for *recB* and 1.6 for *recF* mutants (Figure 2). This suggests that both double strand breaks and single strand lesions are formed on DNA, with a more dramatic effect on double strand DNA breaks. The *recB* mutant also grew more slowly in tobramycin than the *recF* mutant (data not shown).

The presence of double strand (ds) lesions is compatible with the fact that hydroxyl radicals (OH^−^) target DNA and cause ds breaks. The Fenton reaction is the major source of OH^−^ radical formation in the presence of Fe^2+^ ions [12]–[13]. If OH^−^ radicals are responsible for SOS induction, then iron is also expected to be essential for inducing SOS. We used 2,2′-dipyridyl (DP), an iron chelator that prevents the Fenton reaction, to test whether iron depletion affects SOS induction by tobramycin. DP did not change the basal level of fluorescence in LB. In the presence of tobramycin, the SOS induction was decreased from 3 to 1.3 fold upon addition of DP (Figure 2).

Single strand and double strand lesions can occur during mismatch repair. Mismatches are formed when a DNA base is incorporated in a non Watson-Crick manner. This can happen during oxidative stress, which leads to the presence of higher levels of oxidized guanine residues (8-oxo-G). During replication, when the DNA polymerase encounters an 8-oxo-G residue, it pairs it to an adenine instead of a cytosine. The repair of such mismatches involves MutY [37]. MutT is also involved in the response to 8-oxo-G by decreasing its concentration in the nucleotide pool. We over-expressed MutY and MutT in *V. cholerae* from a plasmid. The empty plasmid showed higher SOS induction by tobramycin than the wild type strain without any plasmid. This is also reproducible with other plasmids (not shown), and can be explained by the fact that such high copy plasmids introduce more DNA to replicate and thus more potential for DNA damage and repair. When we over-expressed MutY or MutT, we found that they strongly decreased the SOS induction, from 6.2 fold for the wild type carrying the empty vector plasmid to 1.4 and 1.2 fold respectively, confirming that sub-MIC tobramycin treatment led to incorporation of 8-oxo-G residues (Figure 2).

**Figure 3:**
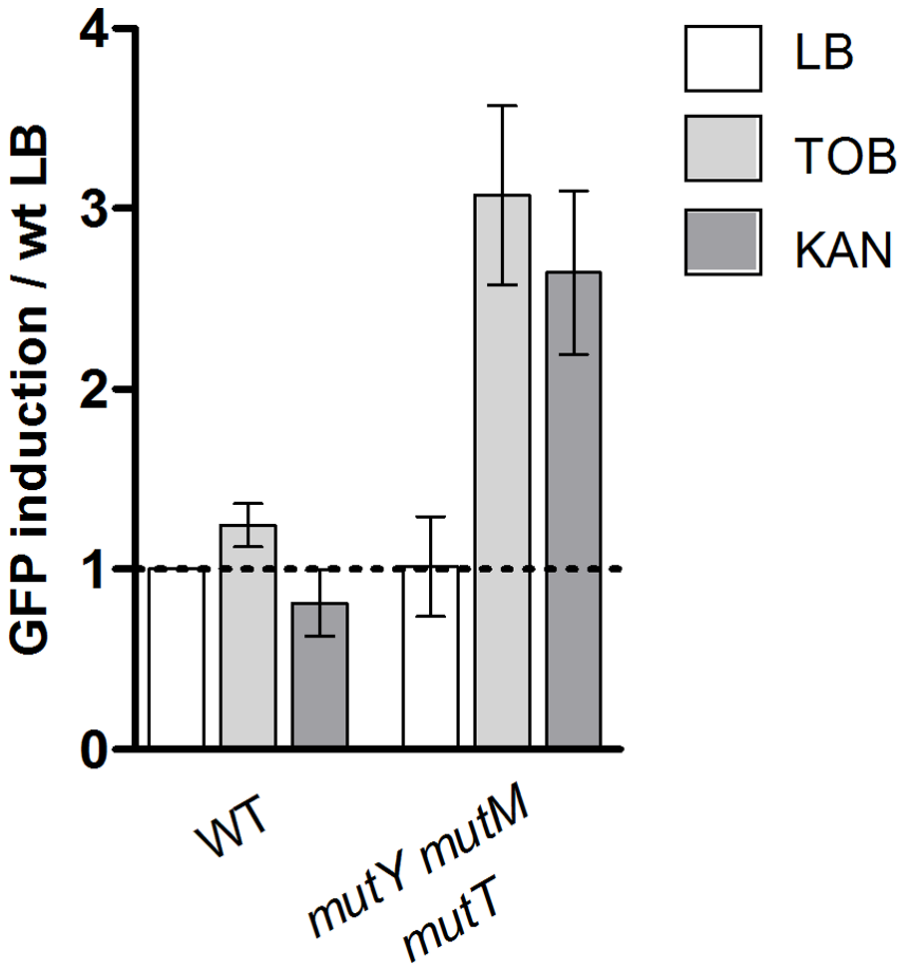
Oxidized guanine incorporation after tobramycin treatment induces SOS in the *E.*coli base excision repair deficient mutant. Histogram bars show the ratio of GFP fluorescence in the presence of antibiotic over fluorescence of the wild type strain grown in LB and thus reflect the induction of SOS. TOB: tobramycin 0.1 µg/ml. KAN: kanamycin 0.2 µg/ml. WT is *E. coli* is BER proficient.

Moreover, when the base excision repair pathway for 8-oxo-G was impaired in *E. coli*, (using the *E. coli ΔmutT ΔmutM ΔmutY* strain), sub-MIC tobramycin and kanamycin (another AG) treatments resulted in SOS response induction, whereas no SOS induction was observed in BER proficient *E. coli*, further implicating the presence of incorporated 8-oxo-G residues as responsible for SOS induction (Figure 3).

Altogether, this first set of results shows that *V. cholerae* is more prone to react to oxidative stress than *E. coli*, forming reactive oxygen species at low doses of antibiotics, which can explain SOS induction by sub-MICs in this species.

In order to understand and clarify the origin of these differences between *E. coli* and *V. cholerae*, we decided to address the role of the RpoS regulon on sub-MIC tobramycin-induced SOS.

### RpoS is involved in the protection of *E. coli* and *V. cholerae* from ROS–induced SOS

It is well established that the RpoS-dependent general stress response is triggered in response to oxidative stress.

Several genes expressed after RpoS regulon induction, namely catalases (KatE, KatG) and iron chelators (Dps), protect cells from ROS related DNA damage [12]–[13], [28]. It has been shown in *Vibrio vulnificus* that RpoS is essential in exponential growth phase to overcome H_2_O_2_ related oxidative stress [29]. We constructed a *V. cholerae* strain deleted for *rpoS*. We performed viability tests and found that the mutant strain did not grow in 2 mM H_2_O_2_ (data not shown), confirming that RpoS is essential for *V. cholerae* growth during oxidative stress.

**Figure 4:**
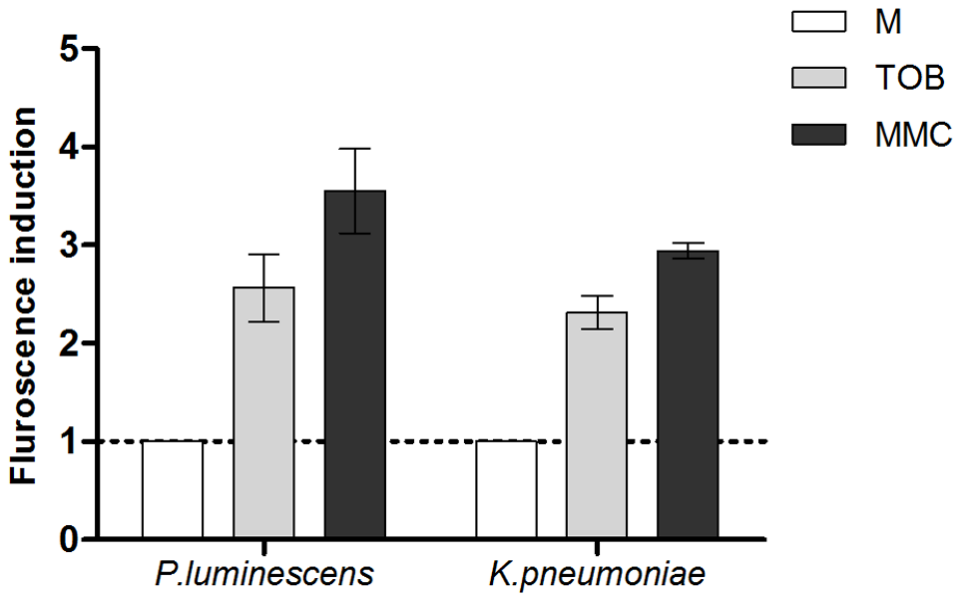
Sub-MIC aminoglycosides induce SOS in other pathogens. Histogram bars show the ratio of GFP fluorescence in the presence of antibiotic over fluorescence in LB and thus reflect the induction of SOS by the antibiotic. Tobramycin (TOB) concentrations were 0.01 µg/ml for *Klebsiella pneumoniae* and 0.0025 µg/ml for *Photorhabdus luminescens*. Mitomycin C (MMC) was used at 0.1 µg/ml.

RpoS is targeted to the ClpXP complex for degradation by the RssB protein. In *E. coli*, IraD binds to and titrates RssB during oxidative stress induction so that less free RssB is present in the cell to bind RpoS. IraD thus protects RpoS from degradation. As mentioned in the introduction, IraD is absent from the genome of a majority of bacterial species, whereas RssB is conserved. In order to address whether the absence of IraD plays a role in the induction of SOS by sub-MIC tobramycin in other species, we decided to test two other unrelated pathogens, *Klebsiella pneumoniae* and *Photorhabdus luminescens*. In order to follow SOS induction in these species, we transformed them, as we did for *E. coli*, with the plasmid p9092 carrying the *Pint-gfp* fusion. We found that sub-MIC tobramycin treatment induced SOS in both species, as it did for *V. cholerae* (Figure 4).

**Figure 5:**
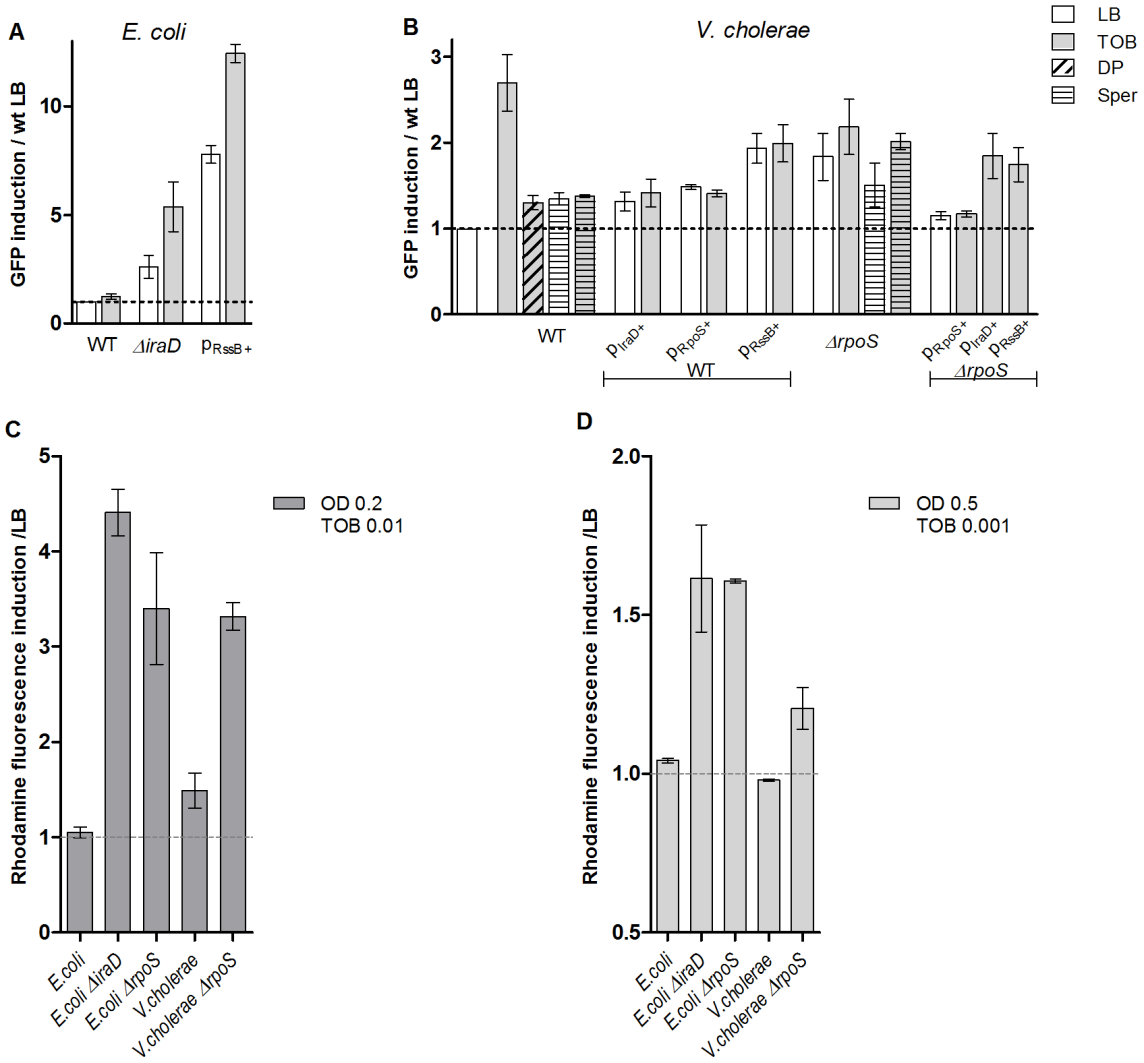
RpoS protects *E.*coli and *V. cholerae* from tobramycin-dependent SOS induction and ROS formation. A and B. Histogram bars show the ratio of GFP fluorescence in the presence of antibiotic over fluorescence of the wild type strain grown in LB and thus reflect the induction of SOS. pRssB+ is pTOPO-RssB+ (pA209). pIraD+ is pTOPO-IraD+ (pA208). pRpoS+ is pTOPO-RpoS+ (pA237). Tobramycin (TOB) concentrations were 0.01 µg/ml for *V. cholerae* and 0.1 µg/ml for *E. coli*. Spermidine (Sper) was used at a final concentration of 10 mM. 2,2 Dipyridil (DP) was used at a final concentration of 0.5 mM. A: *E. coli* p9092. B: *V. cholerae*::p4640. C and D. Histogram bars show the ratio of rhodamine fluorescence in the presence of Tobramycin (TOB). C. Cultures were treated with TOB 0.01 µg/ml and rhodamine fluorescence was measured at OD600 nm 0.2. D. Cultures were treated with TOB 0.001 µg/ml and rhodamine fluorescence was measured at OD600 nm 0.5.

As the *iraD* gene is absent from the *V. cholerae* genome, we hypothesized that higher levels of RpoS are present in the cell during sub-MIC tobramycin treatment in *E. coli*, than in *V. cholerae*, allowing *E. coli* to cope more easily with oxidative stress and avoid eventual DNA damage. In order to test this hypothesis, we first deleted *iraD* in *E. coli*. [32]. We used the *Pint-gfp* fusion (GFP fused to the *intIA* promoter) as a reporter of SOS induction (p9092 for *E. coli*) [2]. *sfiA* is another gene regulated by the SOS response and commonly used for SOS measurement assays (as in [1]). GFP fused to the *sfiA* promoter was also tested and gave the same induction profiles as the *intIA* promoter (not shown). We chose to carry on with the *intIA* promoter in order to have the same reporter promoter in *E. coli* as in *V. cholerae*. IraD protects RpoS from degradation. Deleting *iraD* in *E. coli* MG1655 resulted in SOS induction following sub-MIC tobramycin treatment (Figure 5A). RssB targets RpoS to degradation. Conversely, we found that RssB over-expression strongly induced SOS in these conditions (Figure 5A).

**Figure 6:**
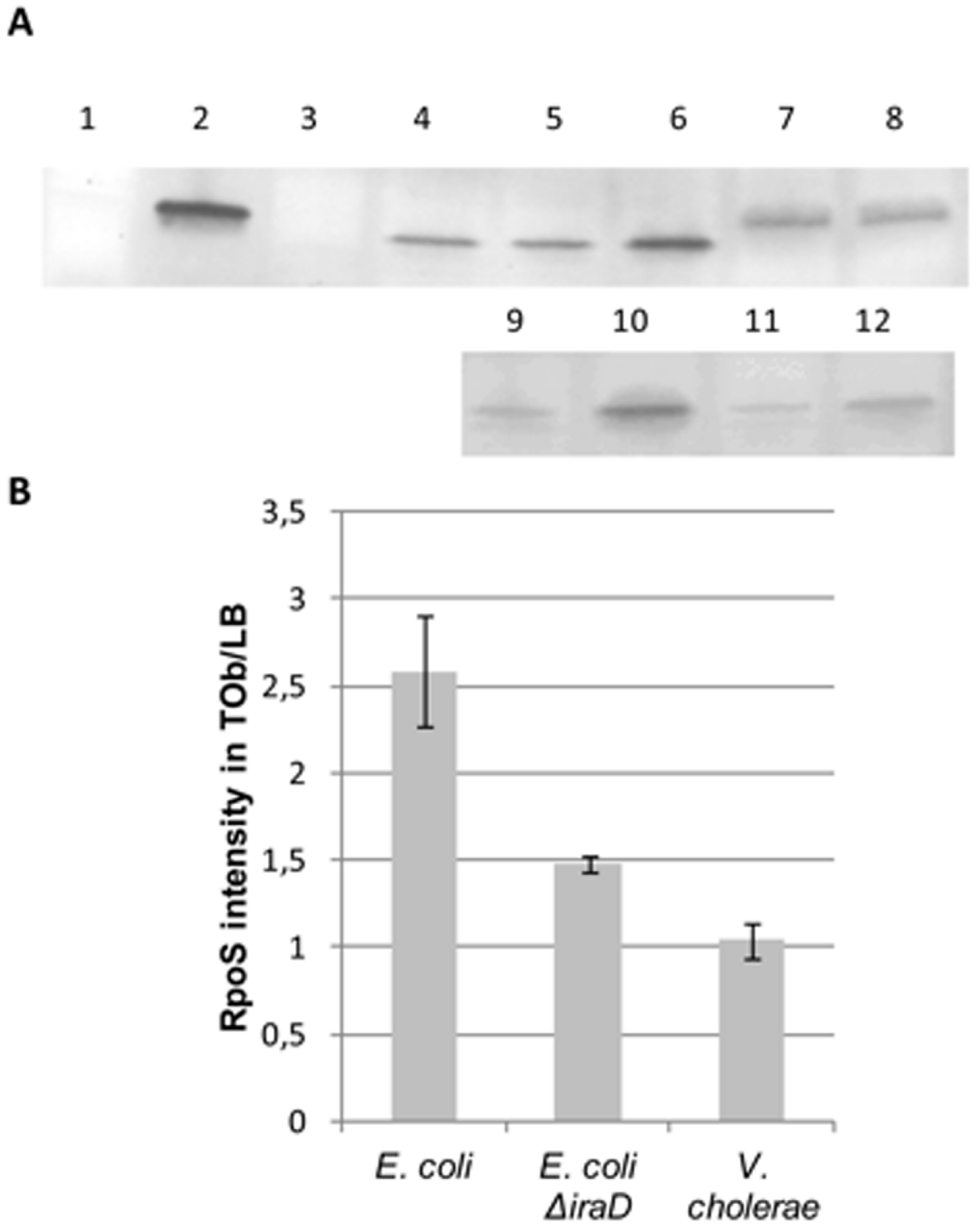
RpoS protein levels are higher in the presence of sub-MIC tobramycin in *E.*coli but unchanged in *V. cholerae*. Overnight cultures were diluted to OD 0.1. Tobramycin treatment at 0.5 µg/ml was initiated at OD 0.5 for one hour. The growth was then stopped and the western blotting was performed. Note that *V. cholerae* RpoS (335 aminoacids) is larger than *E. coli* RpoS (330 aminoacids). A: representative western blots using RpoS antibody. Wells: 1- *V. cholerae ΔrpoS*, 2- Purified *E. coli* RpoS (with His-tag, thus larger than native protein), 3- *E. coli ΔrpoS*, 4- and 9- *E. coli* grown in LB, 5- *E. coli* grown in Tob 0.05 µg/ml, 6- and 10- *E. coli* grown in Tob 0.5 µg/ml, 7- *V. cholerae* grown in LB, 8- *V. cholerae* grown in Tob 0.5 µg/ml, 11- *E. coli ΔiraD* grown in LB, 12- *E. coli ΔiraD* grown in Tob 0.5 µg/ml B: Bands corresponding to RpoS were quantified. The histogram represents the intensity of fluorescence of secondary antibody (Alexa) obtained in tobramycin over the intensity obtained in LB in at least two independent experiments.

We then expressed the *E. coli* anti-adaptor IraD in *V. cholerae* (Figure 5B) and found that in this context sub-MIC tobramycin dependent SOS induction was decreased in *V. cholerae*, whereas SOS induction by MMC was not affected (4 fold induction, not shown on the graph). Over-expression of RpoS also relieved SOS induction following sub-MIC tobramycin treatment. Conversely, when *rpoS* was deleted or when RssB was over-expressed in *V. cholerae*, SOS was significantly induced with or without tobramycin. The higher basal levels when RpoS is low (deletion or high level of RssB) could be a clue to impaired replication in these strains in LB. For instance, when sub-units of DNA polymerase are impaired (such as in *dnaEts* or *dnaNts* mutants at semi-permissive temperature), such SOS induction can also be detected [38]. Over-expression of IraD or RssB in a *V. cholerae ΔrpoS* strain had no effect on SOS, whereas RpoS over-expression complemented efficiently the V. cholerae *ΔrpoS* strain, confirming that the effect observed in the wild type strain is dependent on RpoS (Figure 5B). Western blotting and detection with an RpoS-specific antibody showed that in the presence of tobramycin, the amount of RpoS increased (or RpoS degradation decreased) in wild type *E. coli* and not in wild type *V. cholerae* during exponential phase (Figure 6). Moreover, these increased RpoS amounts were not observed in an *E. coli ΔiraD* strain. Altogether, these data show that protection of RpoS levels is sufficient to relieve sub-MIC AG induced SOS response in *V. cholerae*.

We then addressed whether cellular ROS levels were modified in *E. coli* and *V. cholerae* mutants. We performed the DHR assay on *rpoS* and *iraD* mutants. As the growth of these strains was impaired in tobramycin, instead of measuring fluorescence at OD600 nm 0.5 after TOB 0.01 µg/ml treatment as we did in Figure 1, we either measured fluorescence at the beginning of exponential phase (OD 0.2) for the same concentration of TOB (0.01 µg/ml, Figure 5C), or at the same OD (0.5) for a decreased TOB concentration (0.001 µg/ml, Figure 5D). In both cases, we observed an increase of ROS in *E. coli* and *V. cholerae* when *rpoS* or *iraD* (for *E. coli*) were deleted, showing that decreased RpoS levels lead to ROS formation after TOB treatment in both bacteria.

Finally, it has been observed that addition of polyamines (e.g. putrescine, spermidine) induces RpoS transcription [39] and reduces intracellular ROS production and DNA fragmentation [40]–[41]. Polyamines thus have antioxidant properties and a protective effect on DNA. We tested the effect of spermidine on tobramycin dependent SOS induction in *V. cholerae* wild type or *Δrpos*. We observed that spermidine decreases SOS induction after tobramycin treatment in an RpoS dependent way (Figure 5B).

These results support the idea that RpoS is involved in SOS induction by oxidative stress after AG treatment in *V. cholerae*, and suggest that the RpoS protein level is less stable in *V. cholerae*.

## Discussion

We show here that *V. cholerae* is subject to oxidative stress in response to AG (here tobramycin) at concentrations 100 times below the MIC. These concentrations of AGs seem to lead to the formation of ROS and ultimately to DNA damage through double strand breaks and 8-oxo-G incorporation into DNA. *E. coli* on the other hand, has a stronger resistance to this kind of feeble stress triggered by AGs.

We found that a difference between *V. cholerae* and *E. coli* is that the RpoS sigma factor is protected from degradation by anti-adaptor proteins in *E. coli* upon oxidative stress induction, whereas it seems to be more easily degraded in *V. cholerae*. Indeed, we showed that the protection of *E. coli* RpoS from degradation by the anti-adaptor IraD is important for the protection of the cells against oxidative stress that is triggered in the presence of tobramycin. IraD is part of a genomic island [42]. We looked for other genomes that possess *iraD* orthologs in KEGG (http://www.genome.jp/kegg/) and MicroScope (http://www.cns.fr/agc/microscope/home/index.php) databases containing respectively 2020 and 872 bacterial genomes, including commensal bacteria (like *Bacillus subtilis, Streptococcus agalactiae*) and pathogens such as *Acinetobacter baumannii, Proteus mirabilis, Escherichia coli, Klebsiella pneumoniae, Listeria monocytogenes, Pseudomonas aeruginosa, Streptococcus pneumoniae, Vibrio cholerae, Yersinia pestis* and others. In KEGG, *iraD* orthologs were found in 69 genomes (49 *E. coli* and 5 *Shigella* with 70 to 100% protein identity; 15 *Salmonella* with 40 to 50% identity). In MicroScope, a total of 49 genomes appear to possess *iraD* orthologs where 44 are *E. coli*, 4 *Shigella* and 2 *Salmonella*. No sequenced bacterial organism other than *E. coli*, *Shigella* and *Salmonella* species possess *iraD*, which suggests that *E. coli* is more of an exception than a paradigm for the physiological response to antibiotics sub-MIC. We cannot rule out the possibility that other anti-adaptors exist in *V. cholerae* that are not homologous to IraD. Indeed, the three known *E. coli* anti-adaptors IraD, IraP (responding to phosphate starvation) and IraM (responding to magnesium starvation) do not resemble each other, although they all interact with RssB to protect RpoS from degradation. RpoS levels are also regulated at transcriptional level by *rprA* and *dsrA* small RNAs that bind the *rpoS* mRNA, and protect it from degradation in *E. coli*. Interestingly, like the IraD protein, *rprA* and *dsrA* small RNAs are also absent from *V. cholerae* [31].

**Figure 7:**
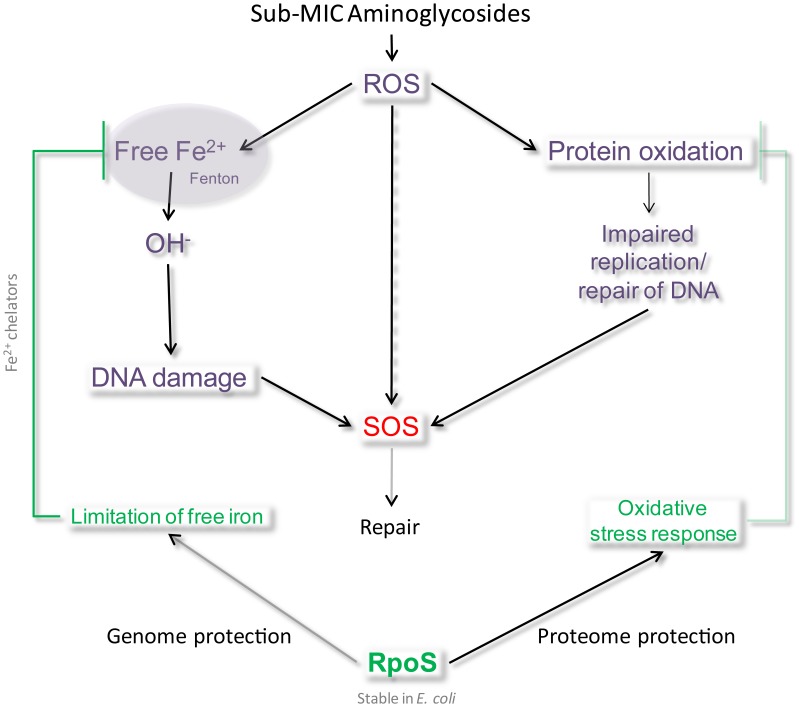
Model of SOS induction by sub-MIC aminoglycosides. We propose that in the presence of sub-MIC AGs, ROS are formed, inducing DNA damage directly (8-oxo-G incorporation) or indirectly (impaired DNA replication and repair proteins). When present and stable, RpoS protects cells from this type of DNA damage by activating an efficient response to oxidative stress, for instance through the decrease of intracellular iron.

Figure 7 represents the model we propose for the induction of SOS after AG treatment: the presence of sub-MIC AG leads to an increase of reactive oxygen species in the bacterial cell, causing oxidative stress. We propose that RpoS levels are insufficient to cope with oxidative stress caused by sub-MIC tobramycin in *V. cholerae*. A more steady presence of RpoS, as in *E. coli*, can lead to more efficient protection from oxidative stress and avoidance of DNA damage. The protective effect of RpoS can be through proteome protection, namely by decreasing the synthesis of iron rich proteins [43] and through genome protection by limiting available free iron and OH^−^ formation [43]–[44]. Mistranslated or misfolded proteins have indeed been shown to be more susceptible to oxidation [19]. Protein oxidation is thus not only a function of ROS availability but also of the levels of aberrant proteins. AGs target the ribosome and cause mistranslation, which could lead to oxidation of mistranslated proteins, and thus an increase in oxidized proteins and eventually DNA damage due to impaired replication and repair. Interestingly, stabilization of a single oxidative stress sensitive protein was shown to be sufficient to enhance oxidative stress resistance of the whole organism in *V. cholerae* [45], confirming the weight of protein oxidation on *V. cholerae*'s ability to cope with stress and RpoS could be a key factor in this process.

Moreover, it was shown in *V. cholerae* that cell envelope damage (caused by chemical treatment, genetic alterations, physical damage) leads to internal oxidative stress, formation of oxygen radicals, changes in iron physiology (increased iron storage) [46] and induces RpoS [46]–[48]. The presence of AGs could be responsible for such envelope stress in *V. cholerae*. RpoS regulates various factors that allow protection from oxidative stress, such as antioxidant agents, but also iron availability sensors like Fur, which prevents iron acquisition when the free iron level is high [43]–[44]. Indeed, excess iron reacts with ROS formed as a natural consequence of aerobic metabolism, and generates hydroxyl radicals through the Fenton reaction. Iron availability is necessary for the toxicity of antibiotics [49]. Another protein induced by RpoS is Dps, a DNA binding iron chelator involved in the protection against killing by bactericidal antibiotics (through hydroxyl radicals) upon oxidative stress during exponential phase [49]. Dps binds and stores iron, which attenuates OH^−^ formation. It is tempting to state here that the effect of RpoS is synergistic with SOS for genome protection, in that RpoS prevents OH^−^ attack on DNA whereas SOS repairs it. Our results using DP are consistent with an effect of iron in the process of DNA damage by sub-MIC tobramycin. So why has *V. cholerae* selected for an RpoS system that is less efficient than that of *E. coli*?

This could be explained by the need for *V. cholerae* to quickly up- and down-regulate the RpoS regulon during infection. Indeed, in contrast to its role in stress response, the effects of RpoS on pathogenesis and virulence are highly variable and depend on bacterial species and their niches [50]. RpoS is conserved within alpha, beta and gamma proteobacteria but the RpoS regulon composition is subject to modification between species [51]. In *Salmonella*, RpoS is essential for virulence. In *E. coli*, RpoS induces virulence genes, but a *ΔrpoS* mutant can outcompete wild type cells in the mouse colon. A tradeoff has been shown between self preservation and nutritional competence in *E. coli* infected patients, determined by levels of RpoS that naturally occur in different *E. coli* cells from the same infecting strain [52]–[53]. In *Vibrio anguillarum*, a fish pathogen, virulence is also up-regulated by RpoS [54]. Conversely, and illustrating the high variability of the RpoS regulon between even closely related species, in *V. cholerae* RpoS represses virulence genes (a *ΔrpoS* strains produces more cholera toxin), and is not required for intestinal survival, even though RpoS contributes to overcoming host-specific stresses in the gut [55]. Nevertheless, RpoS plays an important role in the pathogenicity of *V. cholerae* because it is required for detachment from the epithelial cells after infection and release of bacteria in the environment (the “mucosa escape response”) [56]. This state is characterized by high RpoS levels, RpoS dependent up-regulation of chemotaxis, motility, flagella and reduced virulence gene expression. *V. cholerae* thus has to down-regulate RpoS in order to be virulent in the gut and up-regulate RpoS in order to spread in the environment and survive. This is not the case for *E. coli* where RpoS induces virulence and has no known role in its escape and spread to the environment. The absence of proteins protecting RpoS from degradation in *V. cholerae* could increase the efficiency of the switch between low RpoS (cholera) and high RpoS (spread). Considering this RpoS regulon variability, it was proposed that horizontally transferred genes, which may enhance host adaptation, integrate into the RpoS regulon [51], [57].

In Gram-negative pathogens, acquired multiple antibiotic resistance is in many cases associated with mobile integrons. This natural genetic engineering system is composed of a gene coding an integrase belonging to the site-specific recombinase family and a primary recombination site where gene cassettes can be integrated. The existence of sedentary integron platforms gathering up to 200 gene cassettes in the chromosomes of environmental proteobacteria has been demonstrated [58]–[61]. This is the case for *V. cholerae*, which carries a superintegron gathering more than 170 cassettes [52]. These cassettes can code for antibiotic resistance and other adaptive genes, and their integration by the integrase allows expression from a constitutive promoter upstream of the insertion site [62]–[63]. Moreover, integrase expression is controlled by the SOS response, suggesting that the evolutionary success of these elements largely accounts for the coupling of stress and cassette array remodeling [64]–[65]. We show here that sub-MIC AGs induce SOS and thus integron integrase expression in *V. cholerae*. This AG driven induction, which is apparently common in gamma proteobacteria that lack IraD, likely explains how the numerous cassettes coding for resistance to all AG (43 in 2009 [66]) characterized in mobile integrons were recruited. This implies that the use of these antibiotics may promote cassette rearrangements and expression of integron-borne resistance genes to all families of antibiotics, including the ones that do not induce SOS in *E. coli*.

Bacteria face different growth conditions in different environments. *V. cholerae* has two different niches: the aquatic environment where it grows as a biofilm on crustacean shells where nutrients are scarce, and the host gut, a rich environment where virulence is induced. In both locales, *V. cholerae* may face varying antibiotic concentrations, due to rejections in the environment or antibiotic treatments of the host. Other pathogens are also known to encounter sub-MIC of antibiotics, such as *Pseudomonas aeruginosa*, which causes chronic lung infections where antibiotics subsist as gradients. Strikingly, it was shown that metronidazole treatment of a patient infected with *P. aeroginosa* induced β-lactamase and ceftazidime resistance through SOS-mediated integron rearrangements [67]. It is tempting to make a parallel here with our work with AGs, as neither AGs nor metronidazole cause direct DNA damage, though they are both able to induce the SOS response. It is thus important to better understand how different ecological niches and different life styles modulate evolution of stress responses, which have a major impact on the evolution of genome plasticity and antibiotic resistance, and understanding the molecular mechanisms that drive the emergence of drug resistance can facilitate the design of more effective treatments.

## Materials and Methods

Strain and plasmids used in this study are shown in Table 1, oligonucleotides in Table 2.

**Table 1:** Strains and plasmids.

Strain number	Genotype of interest	Ref. or construction
***E. coli***		
**MG1655**	WT	Laboratory collection
**MK612**	*mutT mutY zrd::tn10 mutM::tn10-km*	[71], Laboratory collection
**A267**	MG1655 *ΔiraD::kan*	This study
**A268**	MG1655 *ΔrpoS::kan*	This study
***V. cholerae***		
**8637**	N16961 *hapR+*	[3]
**A071**	N16961 *hapR+ recF::cm*	This study
**A072**	N16961 *hapR+ recB::cm*	This study
**A321**	N16961 *hapR+ rpoS::aadA1*	This study
**Plasmids**		
**p9644**	pTOPO	Stratagene (circularized)
**p9476**	pTOPO-MutY+	This study
**pA208**	pTOPO-Pbla-IraD+	This study
**pA209**	pTOPO-Pbla-RssB+	This study
**pA237**	pTOPO-Pbla-RpoS+	This study
**p4640** *	*gfp* induced by SOS, SOS reporter for flow cytometry in *V. cholerae*	[1]
**p9092** **	*gfp* induced by SOS, SOS reporter for flow cytometry in *E. coli*	[2]

* p4640 carrying *gfp* fused to the SOS inducible *intIA* promoter was integrated in the chromosome of the specified *V. cholerae* strains by conjugation as described [2].

** p9092, pACYC184 plasmid carrying *gfp* fused to the SOS inducible *intIA* promoter was introduced by heat shock transformation into specified CaCl2 competent *E. coli* strains.

**Table 2:** Oligonucleotides.

Name	Sequence
**693**	TGTGTGGAATTGTGAGCGG
**1598**	ACGGCAGGTATATGTGATGG
**1585**	GTACAAAATACGGTGCGAGCCATTCACTCTTCC
**1586**	CTATTGCCTGCACATTACTAAATCAGAGC
**2041**	GTCAAAATTTGACTAAAAAAGATCCAGTTAAGACGG
**2042**	GCGAGCATCGTTTGTTCGCCCAGCTTCTGTATGGAACGGGAGCGGCCTCCCCCTGGCAACTTTGCGAGTCATTGCGATTTACAACC
**2043**	CGTGAAAGGCGAGATCACCAAGGTAGTCGGCAAATAATGTCTTTTTCCAGACTCATCCAAAACTAAGGCACCCGG
**2044**	CCGAGTGGCTTGCCAAAGAGATTGGTGCC
**2045**	TAAATCGCAATGACTCGCAAAGTTGCCAGGGGGAGGCCGCCCCGTTCCATACAGAAGCTGGGCGAACAAACGATGCTCGC
**2046**	GCACCCCACCGGGTGCCTTAGTTTTGGATGAGTCTGGAAAAAGACATTATTTGCCGACTACCTTGGTGATCTCGCCTTTCACG
**2206**	TGTAAGTTTATACATAGGCGAGTACTCTGTTATGGATGATGCGACAATCACTTCAGG
**2207**	TTAGCTGACATTCTCCAGCGTCG
**2208**	TGTAAGTTTATACATAGGCGAGTACTCTGTTATGGATGACGCAGCCATTGGTCGG
**2209**	TCATTCTGCAGACAACATCAAGCGCAGTCG
**2210**	TGTAAGTTTATACATAGGCGAGTACTCTGTTATGGATGAGTGTCAGCAATACCGTAACC
**2211**	TTAGTTGTCGTATTCGACGTTAAACAGC

### Constructions

#### Strains

A267 was constructed by P1 preparation on strain JW5782-2 11574 (KEIO collection) and transduction in MG1655. A268: P1 preparation on strain JW5437-1 11387 (KEIO collection) and transduction in MG1655. A071: PCR amplification of *recF::cm* using oligonucleotides 1598/693 on plasmid pMEV227 (Marie-Eve Kennedy Val, unpublished) and chitin transformation [2] in strain 8637. A072: PCR amplification of *recB::cm* using oligonucleotides 1598/693 on plasmid pMEV228 (Marie-Eve Kennedy Val, unpublished) and chitin transformation [2] in strain 8637. A321: PCR amplification of regions flanking rpoS using oligonucleotides 2041/2042 and 2043/2044 on strain 8637. PCR amplification of *aadA1* conferring spectinomycin resistance on pAM34 using oligonucleotides 2045/2046 sur. PCR-assembly (as described [2]) of the *rpoS::aadA1* fragment using oligonucleotides 2041/2044 and chitin transformation.

*Klebsiella pneumoniae* was grown in LB medium at 37°C. Electroporation of *Klebsiella pneumoniae* was performed following the same protocol as for *E. coli* using ice-cold 10% glycerol solution. Tetracycline was used at a concentration of 15 µg/ml for the selection of clones carrying plasmid p9092. The MIC of tobramycin was determined using E-tests as 1 µg/ml as for *V. cholerae* so a sub-MIC concentration of 0.01 µg/ml was used for SOS induction assays (repeated 3 times).

*Photorhabdus luminescens* was grown on liquid Schneider Drosophila medium (Sigma) and solid Nutrient Agar (Sigma) at 30°C. Electroporation of *Photorhabdus luminescens* was performed as described [68] using ice-cold SH buffer (5% sucrose 1 mM Hepes). After 3 hours of incubation at 30°C following electroporation, 5 µg/ml tetracycline was added to the culture and incubation over-night at 30°C with shaking before plating on selective medium. Tetracycline was used at a concentration of 10 µg/ml for the selection of clones carrying plasmid p9092. The MIC of tobramycin was determined using E-tests to be 0.25 µg/ml so a sub-MIC concentration of 0.0025 µg/ml was used for SOS induction assays (repeated 3 times).

#### Plasmids

p9476: PCR amplification of *mutY* with its own promoter using oligonucleotides 1585/1586 on strain 8637, cloned in pTOPO-TA. pA208: PCR amplification of *iraD* with the constitutive *bla* promoter using oligonucleotides 2206/2207 on strain MG1655, cloned in pTOPO-TA. pA209: PCR amplification of *rssB* with the constitutive *bla* promoter using oligonucleotides 2208/2209 on strain MG1655, cloned in pTOPO-TA. pA237: PCR amplification of *rpoS* with the constitutive *bla* promoter using oligonucleotides 2210/2211 on strain MG1655, cloned in pTOPO-TA.

#### Detection of intracellular ROS by DHR123

Overnight cultures of *E. coli* MG1655 and *V. cholerae* 8637 were diluted 100 fold in LB or LB+ sub-MIC tobramycin. DHR123 (Sigma) was added to the cultures at a final concentration of 0.9 µg/ml (i.e. 2.5.10^−3^ µM). Cultures were grown to the specified OD 600 nm (OD 0.5 for “exp phase” and OD 0.8 for “end exp phase”). DHR fluorescence was measured at 500 nm excitation wavelength and 550 nm emission wavelength on a TECAN infinite M200. Experiments were repeated at least twice.

#### Flow cytometry

Flow cytometry experiments were performed as described [1], [3] and repeated at least 6 times: briefly, overnight cultures were diluted 100 fold in LB or LB+ sub-MIC antibiotic. Spermidine was used at a final concentration of 10 mM. 2,2 Dipyridil was used at a final concentration of 0.5 mM. After 6 to 8 hours of culture, the culture was washed in PBS and the GFP fluorescence was measured using the Miltenyi MACSQuant device.

#### RpoS immunoblot

Overnight cultures were diluted 100 fold in LB and grown at 37°C. At OD_600_ 0.3, 0.5 µg/ml tobramycin was added to cultures for one hour. Control cultures without tobramycin were also performed. Protein extracts were prepared as previously described [69]. SDS-PAGE was carried out in 4–15% polyacrylamide minigels (Mini Protean II; Bio-Rad). 20 µg of total proteins were loaded into each lane. The proteins were transferred to PVDF membrane with a Trans-Blot turbo (Bio-Rad). Western blots were performed in PBS-T with 1% BSA by incubating the membrane for one hour with a 1/20 000 dilution of polyclonal rabbit antibodies against the RpoS protein [70] and for one hour with a 1/5 000 dilution of Invitrogen Alexa fluor 750 goat anti rabbit antibody. The bands corresponding to RpoS were visualized and quantifyed using an Odyssey. Negative controls were performed with *E. coli* Δ*rpoS* and *V. cholerae* Δ*rpoS* strains. Experiments were repeated twice.

## References

[1] Baharoglu Z, Bikard D, Mazel D (2010) Conjugative DNA transfer induces the bacterial SOS response and promotes antibiotic resistance development through integron activation. PLoS Genet 6: e1001165 doi:10.1371/journal.pgen.1001165. 20975940 10.1371/journal.pgen.1001165 PMC2958807

[2] Baharoglu Z, Krin E, Mazel D (2012) Transformation-induced SOS regulation and carbon catabolite control of the V. cholerae integron integrase: connecting environment and genome plasticity. J Bacteriol 10.1128/JB.05982-11 PMC3302476 22287520

[3] Baharoglu Z, Mazel D (2011) Vibrio cholerae triggers SOS and mutagenesis in response to a wide range of antibiotics, a route towards multi-resistance. Antimicrob Agents Chemother 10.1128/AAC.01549-10 PMC3088271 21300836

[4] Walker GC (1996) The SOS Response of Escherichia coli. Escherichia coli and Salmonella. Neidhardt, FC Washington DC American Society of Microbiology 1: 1400–1416.

[5] Fick J, Soderstrom H, Lindberg RH, Phan C, Tysklind M, et al (2009) Contamination of surface, ground, and drinking water from pharmaceutical production. Environ Toxicol Chem 28: 2522–2527. 19449981 10.1897/09-073.1

[6] Hughes D, Andersson DI (2012) Selection of resistance at lethal and non-lethal antibiotic concentrations. Curr Opin Microbiol 15: 555–560. 22878455 10.1016/j.mib.2012.07.005

[7] Liu YC, Huang WK, Huang TS, Kunin CM (1999) Detection of antimicrobial activity in urine for epidemiologic studies of antibiotic use. J Clin Epidemiol 52: 539–545. 10408993 10.1016/s0895-4356(99)00027-x

[8] Haggard BE, Bartsch LD (2009) Net changes in antibiotic concentrations downstream from an effluent discharge. J Environ Qual 38: 343–352. 19141825 10.2134/jeq2007.0540

[9] Davies J, Spiegelman GB, Yim G (2006) The world of subinhibitory antibiotic concentrations. Curr Opin Microbiol 9: 445–453. 16942902 10.1016/j.mib.2006.08.006

[10] Kohanski MA, DePristo MA, Collins JJ (2010) Sublethal antibiotic treatment leads to multidrug resistance via radical-induced mutagenesis. Mol Cell 37: 311–320. 20159551 10.1016/j.molcel.2010.01.003 PMC2840266

[11] Jena NR (2012) DNA damage by reactive species: Mechanisms, mutation and repair. J Biosci 37: 503–517. 22750987 10.1007/s12038-012-9218-2

[12] Daly MJ (2009) A new perspective on radiation resistance based on Deinococcus radiodurans. Nat Rev Microbiol 7: 237–245. 19172147 10.1038/nrmicro2073

[13] Keyer K, Imlay JA (1996) Superoxide accelerates DNA damage by elevating free-iron levels. Proc Natl Acad Sci U S A 93: 13635–13640. 8942986 10.1073/pnas.93.24.13635 PMC19375

[14] Touati D (2000) Iron and oxidative stress in bacteria. Arch Biochem Biophys 373: 1–6. 10620317 10.1006/abbi.1999.1518

[15] Kuzminov A (1999) Recombinational repair of DNA damage in Escherichia coli and bacteriophage lambda. Microbiol Mol Biol Rev 63: 751–813, table of contents. 10585965 10.1128/mmbr.63.4.751-813.1999 PMC98976

[16] Lu AL, Li X, Gu Y, Wright PM, Chang DY (2001) Repair of oxidative DNA damage: mechanisms and functions. Cell Biochem Biophys 35: 141–170. 11892789 10.1385/CBB:35:2:141

[17] Sobol RW (2012) For MutY, it's all about the OG. Chem Biol 19: 313–314. 22444586 10.1016/j.chembiol.2012.03.002 PMC3891578

[18] Foti JJ, Devadoss B, Winkler JA, Collins JJ, Walker GC (2012) Oxidation of the guanine nucleotide pool underlies cell death by bactericidal antibiotics. Science 336: 315–319. 22517853 10.1126/science.1219192 PMC3357493

[19] Dukan S, Farewell A, Ballesteros M, Taddei F, Radman M, et al (2000) Protein oxidation in response to increased transcriptional or translational errors. Proc Natl Acad Sci U S A 97: 5746–5749. 10811907 10.1073/pnas.100422497 PMC18504

[20] Al Mamun AA, Gautam S, Humayun MZ (2006) Hypermutagenesis in mutA cells is mediated by mistranslational corruption of polymerase, and is accompanied by replication fork collapse. Mol Microbiol 62: 1752–1763. 17427291 10.1111/j.1365-2958.2006.05490.x

[21] Krisko A, Radman M (2010) Protein damage and death by radiation in Escherichia coli and Deinococcus radiodurans. Proc Natl Acad Sci U S A 107: 14373–14377. 20660760 10.1073/pnas.1009312107 PMC2922536

[22] Allen KJ, Griffiths MW (2012) Impact of hydroxyl- and superoxide anion-based oxidative stress on logarithmic and stationary phase Escherichia coli O157:H7 stress and virulence gene expression. Food Microbiol 29: 141–147. 22029928 10.1016/j.fm.2011.09.014

[23] Weber H, Polen T, Heuveling J, Wendisch VF, Hengge R (2005) Genome-wide analysis of the general stress response network in Escherichia coli: sigmaS-dependent genes, promoters, and sigma factor selectivity. J Bacteriol 187: 1591–1603. 15716429 10.1128/JB.187.5.1591-1603.2005 PMC1063999

[24] Merrikh H, Ferrazzoli AE, Bougdour A, Olivier-Mason A, Lovett ST (2009) A DNA damage response in Escherichia coli involving the alternative sigma factor, RpoS. Proc Natl Acad Sci U S A 106: 611–616. 19124769 10.1073/pnas.0803665106 PMC2626751

[25] Merrikh H, Ferrazzoli AE, Lovett ST (2009) Growth phase and (p)ppGpp control of IraD, a regulator of RpoS stability, in Escherichia coli. J Bacteriol 191: 7436–7446. 19820090 10.1128/JB.00412-09 PMC2786607

[26] Battesti A, Tsegaye YM, Packer DG, Majdalani N, Gottesman S (2012) H-NS Regulation of IraD and IraM Anti-adaptors for Control of RpoS Degradation. J Bacteriol 10.1128/JB.00132-12 PMC3347191 22408168

[27] Barth E, Gora KV, Gebendorfer KM, Settele F, Jakob U, et al (2009) Interplay of cellular cAMP levels, {sigma}S activity and oxidative stress resistance in Escherichia coli. Microbiology 155: 1680–1689. 19372151 10.1099/mic.0.026021-0 PMC2848814

[28] Henle ES, Linn S (1997) Formation, prevention, and repair of DNA damage by iron/hydrogen peroxide. J Biol Chem 272: 19095–19098. 9235895 10.1074/jbc.272.31.19095

[29] Park KJ, Kang MJ, Kim SH, Lee HJ, Lim JK, et al (2004) Isolation and characterization of rpoS from a pathogenic bacterium, Vibrio vulnificus: role of sigmaS in survival of exponential-phase cells under oxidative stress. J Bacteriol 186: 3304–3312. 15150215 10.1128/JB.186.11.3304-3312.2004 PMC415748

[30] Hengge-Aronis R (2002) Signal transduction and regulatory mechanisms involved in control of the sigma(S) (RpoS) subunit of RNA polymerase. Microbiol Mol Biol Rev 66: 373–395, table of contents. 12208995 10.1128/MMBR.66.3.373-395.2002 PMC120795

[31] McCullen CA, Benhammou JN, Majdalani N, Gottesman S (2010) Mechanism of positive regulation by DsrA and RprA small noncoding RNAs: pairing increases translation and protects rpoS mRNA from degradation. J Bacteriol 192: 5559–5571. 20802038 10.1128/JB.00464-10 PMC2953674

[32] Bougdour A, Cunning C, Baptiste PJ, Elliott T, Gottesman S (2008) Multiple pathways for regulation of sigmaS (RpoS) stability in Escherichia coli via the action of multiple anti-adaptors. Mol Microbiol 68: 298–313. 18383615 10.1111/j.1365-2958.2008.06146.x

[33] Bougdour A, Wickner S, Gottesman S (2006) Modulating RssB activity: IraP, a novel regulator of sigma(S) stability in Escherichia coli. Genes Dev 20: 884–897. 16600914 10.1101/gad.1400306 PMC1472289

[34] Webb C, Moreno M, Wilmes-Riesenberg M, Curtiss R 3rd, Foster JW (1999) Effects of DksA and ClpP protease on sigma S production and virulence in Salmonella typhimurium. Mol Microbiol 34: 112–123. 10540290 10.1046/j.1365-2958.1999.01581.x

[35] Wilhelm J, Vytasek R, Ostadalova I, Vajner L (2009) Evaluation of different methods detecting intracellular generation of free radicals. Mol Cell Biochem 328: 167–176. 19301099 10.1007/s11010-009-0086-5

[36] Henderson LM, Chappell JB (1993) Dihydrorhodamine 123: a fluorescent probe for superoxide generation? Eur J Biochem 217: 973–980. 8223655 10.1111/j.1432-1033.1993.tb18328.x

[37] Au KG, Clark S, Miller JH, Modrich P (1989) Escherichia coli mutY gene encodes an adenine glycosylase active on G-A mispairs. Proc Natl Acad Sci U S A 86: 8877–8881. 2682664 10.1073/pnas.86.22.8877 PMC298393

[38] Flores MJ, Sanchez N, Michel B (2005) A fork-clearing role for UvrD. Mol Microbiol 57: 1664–1675. 16135232 10.1111/j.1365-2958.2005.04753.x

[39] Igarashi K, Kashiwagi K (2006) Polyamine Modulon in Escherichia coli: genes involved in the stimulation of cell growth by polyamines. J Biochem 139: 11–16. 16428314 10.1093/jb/mvj020

[40] Jung IL, Kim IG (2003) Transcription of ahpC, katG, and katE genes in Escherichia coli is regulated by polyamines: polyamine-deficient mutant sensitive to H2O2-induced oxidative damage. Biochem Biophys Res Commun 301: 915–922. 12589799 10.1016/s0006-291x(03)00064-0

[41] Tkachenko AG, Akhova AV, Shumkov MS, Nesterova LY (2012) Polyamines reduce oxidative stress in Escherichia coli cells exposed to bactericidal antibiotics. Res Microbiol 163: 83–91. 22138596 10.1016/j.resmic.2011.10.009

[42] Huang SH, Chen YH, Kong G, Chen SH, Besemer J, et al (2001) A novel genetic island of meningitic Escherichia coli K1 containing the ibeA invasion gene (GimA): functional annotation and carbon-source-regulated invasion of human brain microvascular endothelial cells. Funct Integr Genomics 1: 312–322. 11793250 10.1007/s101420100039

[43] Guillemet ML, Moreau PL (2008) Fur-dependent detoxification of organic acids by rpoS mutants during prolonged incubation under aerobic, phosphate starvation conditions. J Bacteriol 190: 5567–5575. 18556786 10.1128/JB.00577-08 PMC2519383

[44] Sikora AE, Beyhan S, Bagdasarian M, Yildiz FH, Sandkvist M (2009) Cell envelope perturbation induces oxidative stress and changes in iron homeostasis in Vibrio cholerae. J Bacteriol 191: 5398–5408. 19542276 10.1128/JB.00092-09 PMC2725621

[45] Wholey WY, Jakob U (2012) Hsp33 confers bleach resistance by protecting elongation factor Tu against oxidative degradation in Vibrio cholerae. Mol Microbiol 83: 981–991. 22296329 10.1111/j.1365-2958.2012.07982.x PMC3288485

[46] Mey AR, Wyckoff EE, Kanukurthy V, Fisher CR, Payne SM (2005) Iron and fur regulation in Vibrio cholerae and the role of fur in virulence. Infect Immun 73: 8167–8178. 16299312 10.1128/IAI.73.12.8167-8178.2005 PMC1307094

[47] Mika F, Busse S, Possling A, Berkholz J, Tschowri N, et al (2012) Targeting of csgD by the small regulatory RNA RprA links stationary phase, biofilm formation and cell envelope stress in Escherichia coli. Mol Microbiol 10.1111/j.1365-2958.2012.08002.x PMC3465796 22356413

[48] Laubacher ME, Ades SE (2008) The Rcs phosphorelay is a cell envelope stress response activated by peptidoglycan stress and contributes to intrinsic antibiotic resistance. J Bacteriol 190: 2065–2074. 18192383 10.1128/JB.01740-07 PMC2258881

[49] Calhoun LN, Kwon YM (2011) The ferritin-like protein Dps protects Salmonella enterica serotype Enteritidis from the Fenton-mediated killing mechanism of bactericidal antibiotics. Int J Antimicrob Agents 37: 261–265. 21295952 10.1016/j.ijantimicag.2010.11.034

[50] Dong T, Schellhorn HE (2010) Role of RpoS in virulence of pathogens. Infect Immun 78: 887–897. 19948835 10.1128/IAI.00882-09 PMC2825926

[51] Santos-Zavaleta A, Gama-Castro S, Perez-Rueda E (2011) A comparative genome analysis of the RpoS sigmulon shows a high diversity of responses and origins. Microbiology 157: 1393–1401. 21310789 10.1099/mic.0.042937-0

[52] Levert M, Zamfir O, Clermont O, Bouvet O, Lespinats S, et al (2010) Molecular and evolutionary bases of within-patient genotypic and phenotypic diversity in Escherichia coli extraintestinal infections. PLoS Pathog 6: e1001125 doi:10.1371/journal.ppat.1001125. 20941353 10.1371/journal.ppat.1001125 PMC2947995

[53] Rozen DE, Philippe N, Arjan de Visser J, Lenski RE, Schneider D (2009) Death and cannibalism in a seasonal environment facilitate bacterial coexistence. Ecol Lett 12: 34–44. 19019196 10.1111/j.1461-0248.2008.01257.x

[54] Ma L, Chen J, Liu R, Zhang XH, Jiang YA (2009) Mutation of rpoS gene decreased resistance to environmental stresses, synthesis of extracellular products and virulence of Vibrio anguillarum. FEMS Microbiol Ecol 70: 130–136. 19527291 10.1111/j.1574-6941.2009.00713.x

[55] Merrell DS, Tischler AD, Lee SH, Camilli A (2000) Vibrio cholerae requires rpoS for efficient intestinal colonization. Infect Immun 68: 6691–6696. 11083783 10.1128/iai.68.12.6691-6696.2000 PMC97768

[56] Nielsen AT, Dolganov NA, Otto G, Miller MC, Wu CY, et al (2006) RpoS controls the Vibrio cholerae mucosal escape response. PLoS Pathog 2: e109 doi:10.1371/journal.ppat.0020109. 17054394 10.1371/journal.ppat.0020109 PMC1617127

[57] Chiang SM, Schellhorn HE (2010) Evolution of the RpoS regulon: origin of RpoS and the conservation of RpoS-dependent regulation in bacteria. J Mol Evol 70: 557–571. 20506020 10.1007/s00239-010-9352-0

[58] Mazel D, Dychinco B, Webb VA, Davies J (1998) A distinctive class of integron in the Vibrio cholerae genome. Science 280: 605–608. 9554855 10.1126/science.280.5363.605

[59] Rowe-Magnus DA, Guerout AM, Biskri L, Bouige P, Mazel D (2003) Comparative analysis of superintegrons: engineering extensive genetic diversity in the Vibrionaceae. Genome Res 13: 428–442. 12618374 10.1101/gr.617103 PMC430272

[60] Rowe-Magnus DA, Guerout AM, Ploncard P, Dychinco B, Davies J, et al (2001) The evolutionary history of chromosomal super-integrons provides an ancestry for multiresistant integrons. Proc Natl Acad Sci U S A 98: 652–657. 11209061 10.1073/pnas.98.2.652 PMC14643

[61] Rowe-Magnus DA, Guerout AM, Mazel D (2002) Bacterial resistance evolution by recruitment of super-integron gene cassettes. Mol Microbiol 43: 1657–1669. 11952913 10.1046/j.1365-2958.2002.02861.x

[62] Jove T, Da Re S, Denis F, Mazel D, Ploy MC (2010) Inverse correlation between promoter strength and excision activity in class 1 integrons. PLoS Genet 6: e1000793 doi:10.1371/journal.pgen.1000793. 20066027 10.1371/journal.pgen.1000793 PMC2791841

[63] Mazel D (2006) Integrons: agents of bacterial evolution. Nat Rev Microbiol 4: 608–620. 16845431 10.1038/nrmicro1462

[64] Cambray G, Sanchez-Alberola N, Campoy S, Guerin E, Da Re S, et al (2011) Prevalence of SOS-mediated control of integron integrase expression as an adaptive trait of chromosomal and mobile integrons. Mob DNA 2: 6. 21529368 10.1186/1759-8753-2-6 PMC3108266

[65] Guerin E, Cambray G, Sanchez-Alberola N, Campoy S, Erill I, et al (2009) The SOS response controls integron recombination. Science 324: 1034. 19460999 10.1126/science.1172914

[66] Partridge SR, Tsafnat G, Coiera E, Iredell JR (2009) Gene cassettes and cassette arrays in mobile resistance integrons. FEMS Microbiol Rev 33: 757–784. 19416365 10.1111/j.1574-6976.2009.00175.x

[67] Hocquet D, Llanes C, Thouverez M, Kulasekara HD, Bertrand X, et al (2012) Evidence for induction of integron-based antibiotic resistance by the SOS response in a clinical setting. PLoS Pathog 8: e1002778 doi:10.1371/journal.ppat.1002778. 22719259 10.1371/journal.ppat.1002778 PMC3375312

[68] Bennett HP, Clarke DJ (2005) The pbgPE operon in Photorhabdus luminescens is required for pathogenicity and symbiosis. J Bacteriol 187: 77–84. 15601690 10.1128/JB.187.1.77-84.2005 PMC538804

[69] Ruiz N, Silhavy TJ (2003) Constitutive activation of the Escherichia coli Pho regulon upregulates rpoS translation in an Hfq-dependent fashion. J Bacteriol 185: 5984–5992. 14526009 10.1128/JB.185.20.5984-5992.2003 PMC225030

[70] Coynault C, Robbe-Saule V, Norel F (1996) Virulence and vaccine potential of Salmonella typhimurium mutants deficient in the expression of the RpoS (sigma S) regulon. Mol Microbiol 22: 149–160. 8899717 10.1111/j.1365-2958.1996.tb02664.x

[71] Tajiri T, Maki H, Sekiguchi M (1995) Functional cooperation of MutT, MutM and MutY proteins in preventing mutations caused by spontaneous oxidation of guanine nucleotide in Escherichia coli. Mutat Res 336: 257–267. 7739614 10.1016/0921-8777(94)00062-b

